# Genome-Wide Analysis and Expression Profiles of the Dof Family in *Cleistogenes songorica* under Temperature, Salt and ABA Treatment

**DOI:** 10.3390/plants10050850

**Published:** 2021-04-23

**Authors:** Penglei Wang, Zhuanzhuan Yan, Xifang Zong, Qi Yan, Jiyu Zhang

**Affiliations:** The State Key Laboratory of Grassland Agro-Ecosystems, Key Laboratory of Grassland Livestock Industry Innovation, Ministry of Agriculture and Rural Affairs, College of Pastoral Agriculture Science and Technology, Lanzhou University, Lanzhou 730020, China; wangpl18@lzu.edu.cn (P.W.); yanzhzh16@lzu.edu.cn (Z.Y.); zongxf15@lzu.edu.cn (X.Z.); yanq16@lzu.edu.cn (Q.Y.)

**Keywords:** *Cleistogenes songorica*, DNA-binding, expression profile, one zinc finger, phylogenetic analysis, RNA-Seq

## Abstract

The DNA-binding with one zinc finger (*Dof*) family of plant-specific transcription factors has a variety of important functions in gene transcriptional regulation, development, and stress responses. However, the structure and expression patterns of *Dof* family have not been identified in *Cleistogenes songorica*, which is an important xerophytic and perennial gramineous grass in desert grassland. In this study, 50 *Dof* genes were identified in *C. songorica* and could be classified into four groups. According to genome-wide analysis, 46 of 50 *Dof* genes were located on 20 chromosomes, and the gene structure and conserved protein motif of these proteins were analyzed. In addition, phylogenetic analysis of *Dof* genes in *C. songorica*, *Arabidopsis thaliana*, *Oryza sativa*, and *Brachypodium distachyon* estimated the evolutionary relationships, and these genes were grouped into seven clusters. Moreover, the expression profiles of these *Dof* genes in *C. songorica* were analyzed in response to high/low temperature, salinity, and ABA treatments. These results will provide valuable information for future studies on gene classification, cloning, and functional characterization of this family in *C. songorica*.

## 1. Introduction

Transcription factors (TFs) are a group of proteins with important roles in controlling cell activities, such as physiological balance, biochemical reactions, and responses to the environment [1,2,3]. They determine the transcription rate of genes by binding the cis-regulatory elements of promoters [4], and regulate interactions of proteins in a complex network [5,6]. The DNA-binding with one finger (*Dof*) TFs is a representative of the plant-specific transcription factor gene family, which is closely related to physiological and biochemical reactions during plant growth and development [7,8]. The structures and functions of the *Dof* family have been reported and revealed that the Dof proteins have a highly conserved DNA-binding domain at the N-terminal [9,10]. The domain is composed of 52 amino acid residues, including C2-C2 type zinc finger motif, which could recognize the specific regulatory elements of AAAG or CTTT in the promoters of target genes [11,12].

Based on genome-wide analysis, the *Dof* gene family has been identified in many species. 36 *Dof* genes have been identified in *Arabidopsis* [7], 30 in *O. sativa* [13], 27 in *Brachypodium distachyon* [14], 46 in maize [15], 45 in cassava *(Manihot esculenta)* [16], 33 in pepper [17], 74 in banana [18], 42 in *Medicago truncatula* [19], 48 in *Triticum aestivum* [20], 78 in soybean [21], 35 in potato [22], 26 in moso bamboo [23], and 36 in cucumber [9]. It has been reported that the Dof transcription factors have diverse functions that are unique to plant growth and development in many species, such as carbon metabolism in maize [24] and flowering time in *Arabidopsis* [25]. It is also related to the influence of nitrogen in wheat [26], vascular system development and functioning in *Arabidopsis* [27], circadian cycle [28], shoot branching and seed coat formation in *Arabidopsis* [29], and responses to drought and salt stress and flowering-time control in a gene-specific manner [30,31]. Some Dof proteins were functionally characterized in *Arabidopsis*, such as OBF binding protein (OBP1, OBP2, and OBP3), Cycling *Dof* Factors (CDF1-5) and *Dof* Affecting Germination (DAG1 and DAG2) [25,32,33]. For example, OBP3 has been characterized as a novel light signaling component [34]. *MdDof24* was involved in the flower development pathways and some chemical reactions [35].

*Cleistogenes songorica* is a perennial forage, which can grow in saline and desert areas where mean annual rainfall is 110 mm [36]. It is one of the most important native plants in the north-west of China and plays important roles in animal husbandry and pasture production [37]. Many studies have focused on the physiology of *C. songorica.* Previous studies have shown that drought, cold, and high salinity environments could influence important proteins of *C. songorica* and induced genes expression, such as *CsLEA*, *CsSAMS1*, and *CsALDH12A1* [38,39,40] Although there have been some studies on the transcription factor of *bZIP* [3], there is no report on *Dof* transcription factors that may play an important role in surviving abiotic stress in *C. songorica*. The *Dof* genes, especially, involved in the regulation and adjustment of the metabolism under temperature, salt, and ABA, has been described in different plants [17,18,23]. In the present study, we identified all potential *Dof* genes in the *C. songorica* genome. In addition, the biochemical indexes, subcellular localization, chromosome distribution, gene structure, conserved motifs, and phylogenetic analysis of the *C. songorica Dof* family were examined. Finally, we analyzed the gene expression in different response to abiotic stress. These results will provide valuable information for future studies about the functions of the *Dof* family in *C. songorica.*

## 2. Results

### 2.1. Identification and Structure Analysis of Dof Genes

**Figure 1:**
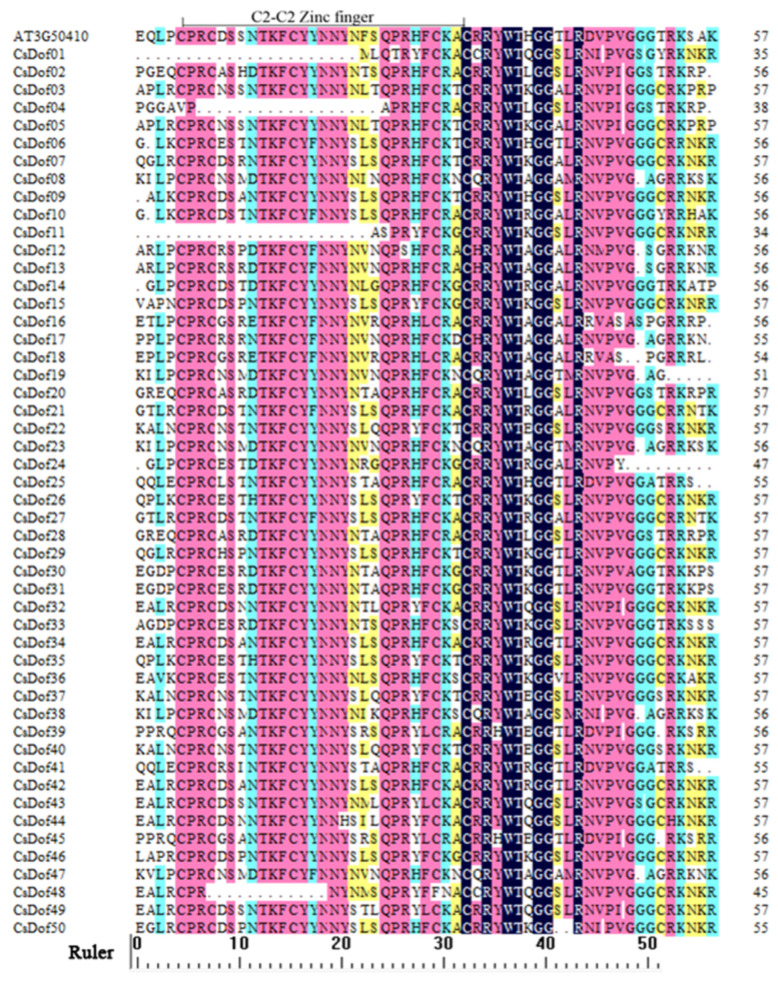
Multiple sequence alignment of the *Dof* domain in *C. songorica* and *Arabidopsis*.

In this study, a total of 50 *Dof* genes were identified in the *C. songorica* genome database [41]. For convenience, these *C. songorica Dof* genes were named *CsDof01*-*CsDof50* (Table S1). The full length of the *CsDof* CDS ranged from 421 bp (*CsDof24*) to 2289 bp (*CsDof10*), with an average length of 1017 bp. The size of the *Dof* proteins varied between 138 (*CsDof24*) and 726 (*CsDof10*) amino acids (aa) with an average of 337 aa. The molecular weight (Mw) ranged from 14628.54 to 78418.04 Da, and the theoretical pI varied from 5.22 (*CsDof19*) to 11.06 (*CsDof03*). Moreover, predictive analysis of subcellular localization revealed that more than 60% of the 50 *CsDof* genes were located in the nucleus (Table S1). SMART and Pfam were used to confirm the conserved *Dof* domain of each sequence according to the protein sequences of these genes. The results showed that most of the *CsDof* genes contained the highly conserved *Dof* domain and constituted the C2-C2 zinc finger structure (Figure 1).

### 2.2. Chromosomal Localization, Gene Duplication, and Gene Structure of CsDof Gene

**Figure 2:**
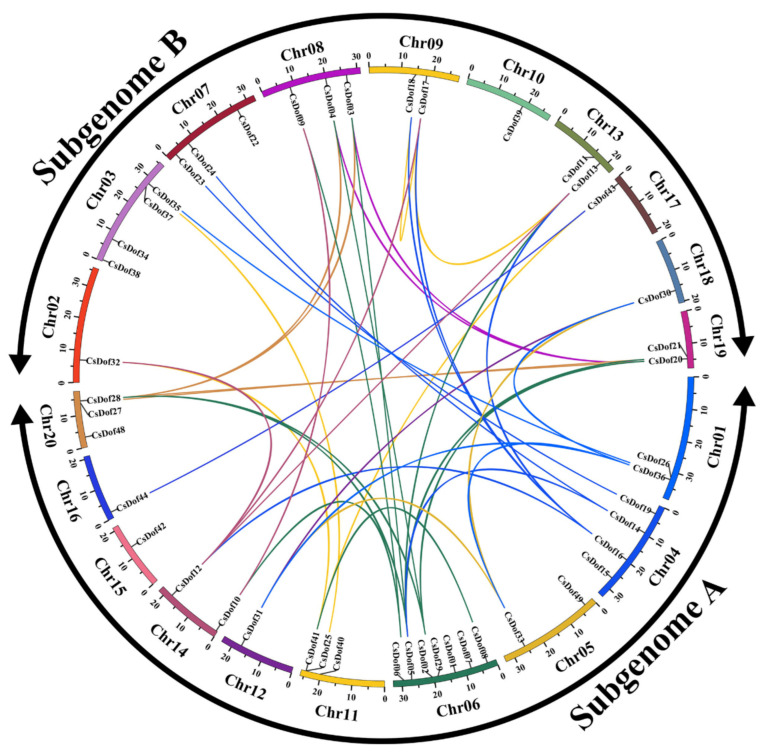
Distribution of the *CsDof* duplicated genes in *C. songorica.* The colored lines within the chromosomes represent the syntentic relationships in *C. songorica* Dof members.

The TBtools software was used to draft the *CsDof* chromosomal location map to show the distribution of each *CsDof* gene on the *C. songorica* chromosome (Figure 2). Referring to the previous study [41], the 20 chromosome of heterotetraploid *C. songorica* genome were divided into 2 subgenomes (Table S1). The results showed that 46 of the 50 *CsDof* genes were located on the 20 chromosomes; however, 4 members (*CsDof45*, *CsDof46*, *CsDof47*, and *CsDof50*), which were anchored on the scaffolds, could not be located on any *C. songorica* chromosomes. The largest number of *CsDof* genes was identified on chromosome 6 (7 genes), followed by chromosomes 3 and 4 (4 genes). Only one *CsDof* gene was located on each of chromosomes 3, 10, 12, 15, 16, 17, and 18.

As shown in Table S2, the *ka/ks* values of 41 pairs duplicated gene smaller than 0.5, and the approximate date of gene duplication events was calculated according to the *Ks* values. This implied that most of the *Dof* duplicated gene pairs tended to be subjected to purifying selection. For the paralogous group *CsDof19*/*CsDof23*, the date of gene duplication events was the latest as 9.02 Mya, while the date of gene duplication events for the paralogous group *CsDof05*/*CsDof21* was earliest as 293.75 Mya. There were 41 pairs of duplicated genes were found in 33 *CsDof* genes (Figure 2). These duplicated genes were the most common on chromosomes 6, 4, and 8, whereas there were zero duplicated gene pairs on chromosomes 10, 15 and one on 2, 12, 16, 17, 18.

**Figure 3:**
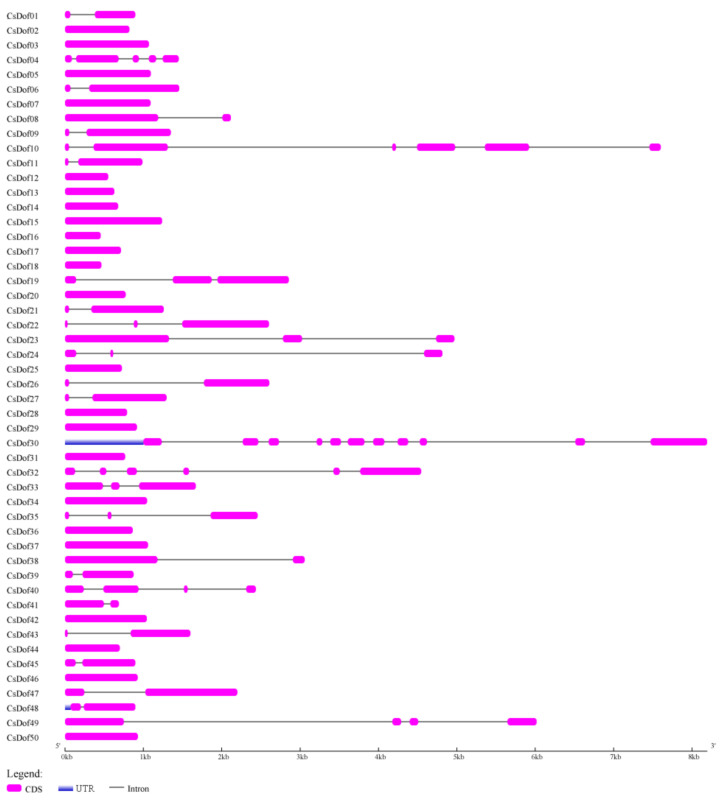
Gene structure of 50 *CsDof* genes in *C. songorica*. CDS, UTR, and introns are indicated by purple, blue, and black, respectively.

To gain further insight into the structural diversity of *CsDof* genes, the exon-intron structures of 50 *CsDof* genes were analyzed. As shown in Figure 3, the number in introns of *CsDof* genes ranged from 0 to 10. Among 50 *CsDof* genes, 23 of the *CsDof* genes were intronless (46%), 15 genes contained only 1 intron (30%), 6 genes contained 2 introns (12%), 2 genes contained 3 introns (4%), and 2 genes contained 5 introns (4%). In addition, *CsDof04* and *CsDof30* contained 4 and 10 introns (2%), respectively.

### 2.3. Conserved Motif, Phylogenetic Analysis, and Classification of the Dof Transcription Factor Family

**Figure 4:**
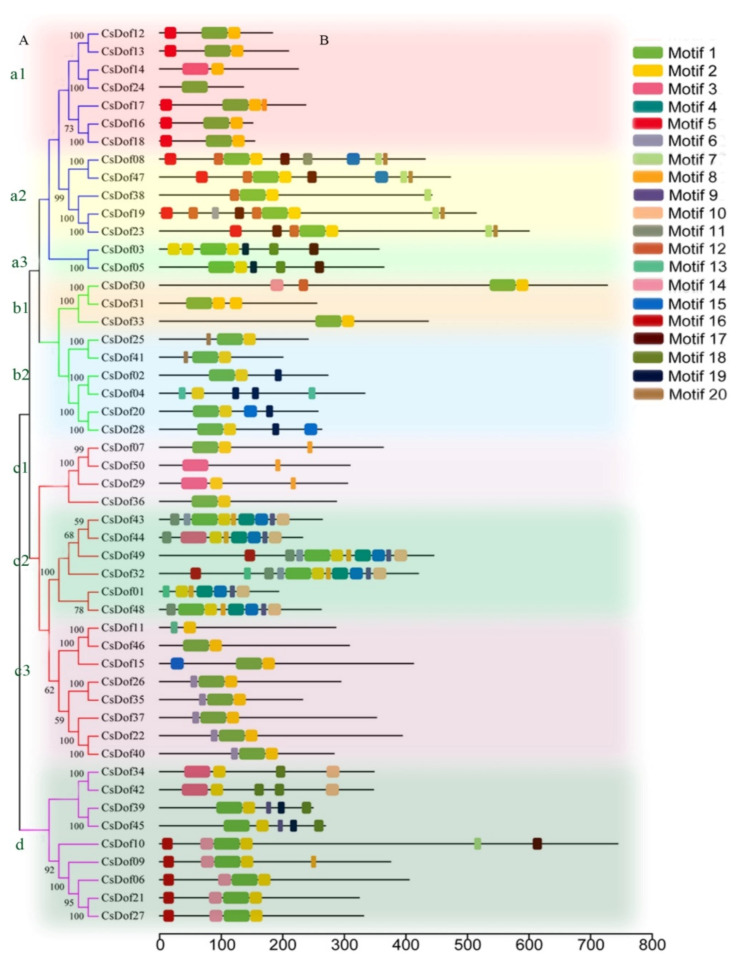
(**A**) Phylogenetic analysis of the *CsDof* genes. The phylogenetic tree was constructed using the NJ method (1000 bootstrap replicates). (**B**) Distribution of conserved motifs in the *CsDof* proteins. Conserved motifs were identified using the MEME program and indicated in numbered colored boxes, where Motif 1, Motif 3, and Motif 2 include the conserved Dof domain. The blue, green, red, and pink branches represent groups a, b, c, and d, respectively. Groups a, b, and c were further divided into subgroups a1, a2, a3, b1, b2, c1, c2, and c3.

The motif was analyzed by the MEME search tool to reveal the diversity of the *Dof* genes in *C. songorica*. In total, 20 motifs, which were named motifs 1–20, were identified in the 50 *Dof* proteins (Figure 4B). Among these motifs, motif 1 and motif 3 have the C2-C2 Zinc finger (Figure S1). Except for *CsDof24* and *CsDof50*, motif 2 was observed in all of the *CsDof* proteins. Most of the *CsDof* proteins contained motif 1 except *CsDof14*, *CsDof04*, *CsDof50*, *CsDof29*, *CsDof44*, *CsDof01*, *CsDof11*, *CsDof34*, and *CsDof42*. As expected, most of the closely related members had common motif compositions within the same subfamily, such as *CsDof10* and *CsDof09* or *CsDof06*, *CsDof21*, and *CsDof27*. The details of these motif features are shown in Figure S1.

The phylogenetic tree based on the 50 *CsDof* amino acid sequences was used to evaluate the evolutionary relationships among *CsDof* proteins by the neighbor-joining method. As shown in Figure 4A, the 50 *CsDof* genes could be divided into four major groups (a, b, c, d). Group c had the most members (18 genes), followed by group a, which contained 14 *CsDof* genes. Group b and group d contained the fewest members 9 *CsDof* genes each. In addition, some *CsDofs* showed similar exon-intron structure patterns within the same group. For example, *CsDof07*, *CsDof15*, *CsDof29*, *CsDof36*, *CsDof37*, *CsDof44*, *CsDof46*, and *CsDof50* in group c had no intron. In group d, all the *CsDof* genes contained one intron, except for *CsDof10*, *CsDof34*, and *CsDof42*.

**Figure 5:**
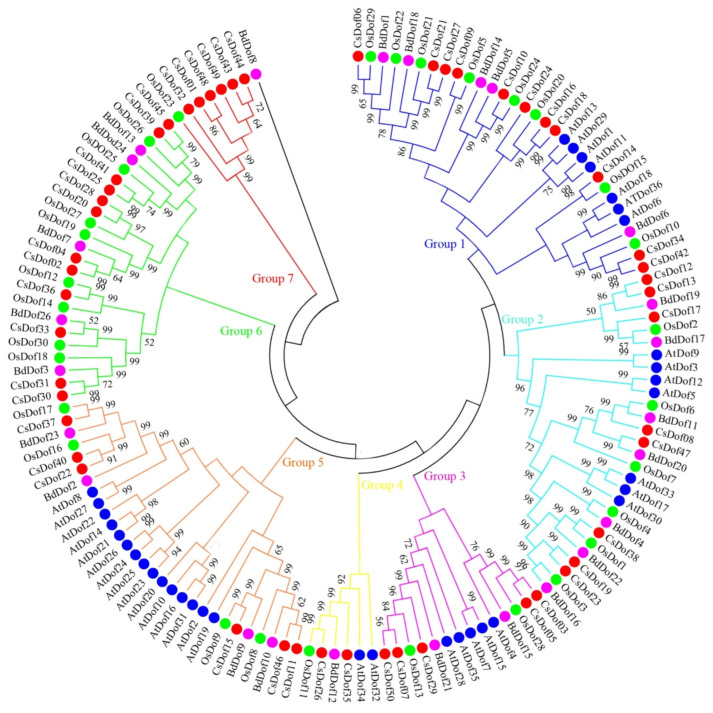
Phylogenetic tree representing relationships among *Dof* domains in *C. songorica*, *A. thaliana*, *O. sativa*, and *B. distachyon*. The different colors indicate different species: red represents *Dof* genes from *C. songorica*, blue represents *Dof* genes from *Arabidopsis*, green represents *Dof* genes from *O. sativa* and purple represents *Dof* genes from *B. distachyon*. Bootstrapping values are indicated as percentages (when >50%) along the branches.

To further estimate the evolutionary relationships among the *C. songorica CsDof* proteins and *Dof* proteins from other species, the *Dof* genes of *C. songorica*, *A. thaliana*, *O. sativa*, and *B. distachyon* were used to construct a phylogenetic tree (Figure 5, Table S4). The phylogenetic tree showed that *Dof* family could be divided into seven groups. Among these groups, group 1 contained the largest number of *Dof* genes (42 members), followed by group 5 (29 members), group 2 (28 members), group 6 (25 members), and group 3 (14 members). Group 4 and group 7 contained the fewest *Dof* genes, including only 6 and 7 members, respectively. There were no *AtDof* genes in group 7 and 6. Group 7 contained seven *Dof* genes, including six *CsDof* genes and one *OsDof* gene.

### 2.4. Expression Profiles of Dof Genes in C. songorica

**Figure 6:**
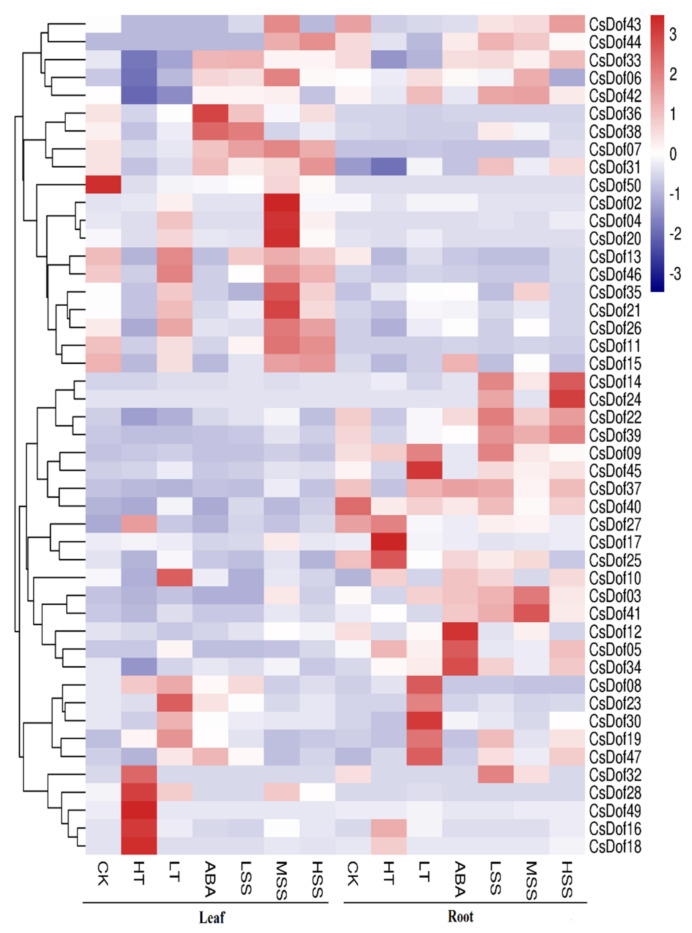
Heat map representation of *CsDof* genes across different treatments in leaf and root at 0 h and 24 h. CK: denotes 0 h. HT: high temperature (40 °C), LT: low temperature, ABA: ABA (100 μM), LSS: 50 mM NaCl (light salt stress), MSS: 100 mM NaCl (moderate salt stress), and HSS: 200 mM NaCl (high salt stress), respectively. The color scale represents FPKM value.

In this study, RNA-seq data was used to study the gene expression profiles. We analyzed the genes expression in the leaves and roots under high temperature (40 °C), low temperature (4 °C), ABA (100 μM), and salt (50 mM, 100 mM and 200 mM) at 0 h and 24 h. As a result, *CsDof01, CsDof24*, *CsDof29*, *CsDof32*, and *CsDof48* had hardly any expression under any treatment. *CsDof43* and *CsDof44* showed no expression in the leaf, and the genes *CsDof07*, *CsDof20*, *CsDof28*, and *CsDof50* showed no expression in the root. On the contrary, most of the *CsDof* genes showed high level of expression in leaves and roots under different treatments; for instance, *CsDof02*, *CsDof04*, *CsDof14*, *and CsDof50* (Table S5, Figure 6).

**Figure 7:**
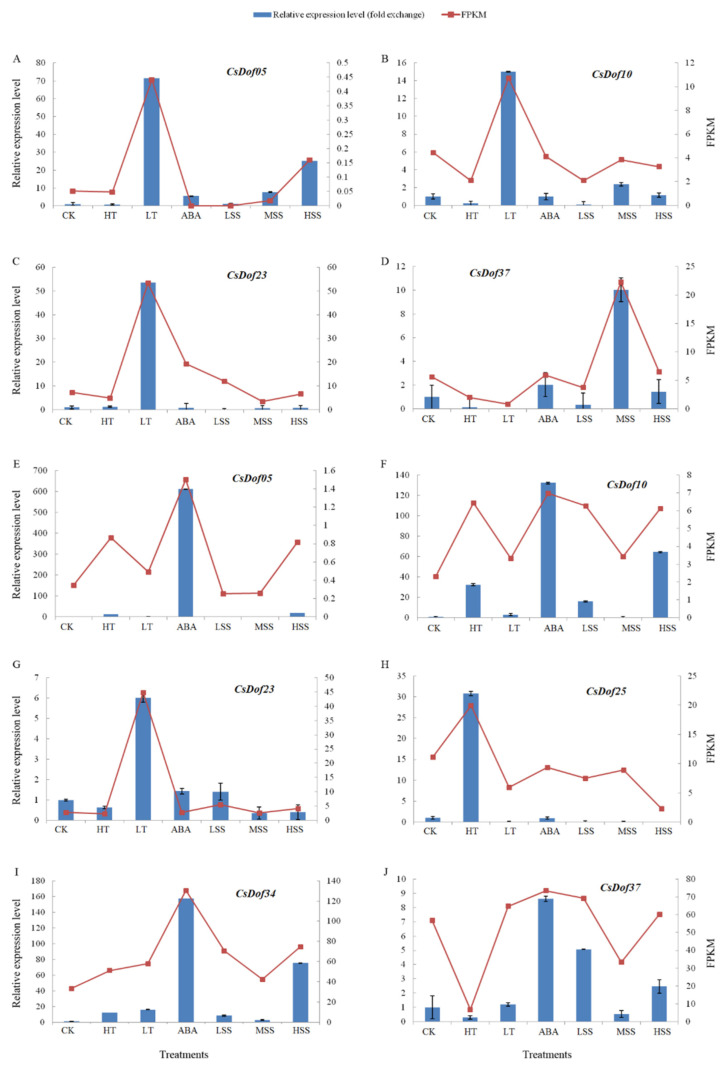
The expression profiles of *CsDof* genes in leaf and root in *C. songorica* under high/low temperature, ABA, and salt stresses. The expression profiles of *CsDof05*, *CsDof10*, *CsDof23*, and *CsDof37* in leaf are shown in (**A**–**D**), respectively. The expression profiles of *CsDof05*, *CsDof10*, *CsDof23*, *CsDof25*, *CsDof34*, and *CsDof37* in root are shown in (**E**–**J**), respectively. The x-axis indicates treatments. Left y-axis indicates relative expression level. Right y-axis means genes abundance change based on FPKM values according to RNA-Seq. Error bars indicate standard deviations of independent biological replicates (*n* = 3). CK: denotes 0 h, HT: high temperature (40 °C), LT: low temperature, ABA: ABA (100 μM), LSS: 50 mM NaCl (light salt stress), MSS: 100 mM NaCl (moderate salt stress), and HSS: 200 mM NaCl (high salt stress), respectively, administered for 24 h.

To gain some insight into the putative role of these proteins, 6 *CsDof* genes were selected for the detection of expression profiles by qPCR under high/low temperature, ABA, and salt treatments. The 6 *CsDof* genes (*CsDof05*, *CsDof10*, *CsDof23*, *CsDof25, CsDof34*, *CsDof37*) were selected from different subgroups for expression analysis [31]. The expression profiles of the *CsDof* genes in the leaf are shown in Figure 7A–D and those in the root are shown in Figure 7E–J. As shown in Figure 7A,B, the *CsDof05* and *CsDof10* genes were upregulated at low temperature, salt (100 mM and 200 mM), and ABA treatments. The *CsDof23* gene was clearly upregulated by low temperature, and downregulated by salt (50 mM) according to Figure 7C. However, the *CsDof37* gene was not expressed at low temperature and was upregulated by salt (50 mM) in Figure 7D, and the date of *CsDof34* and *CsDof25* were not shown in the Figure 7 because they were not expressed under all treatments in the leaf. In the root, the *CsDof05*, *CsDof10*, *CsDof25*, and *CsDof34* genes were upregulated by heat. The *CsDof23* and *CsDof34* genes were upregulated by low temperature. In addition, the *CsDof05*, *CsDof10*, *CsDof34*, and *CsDof37* genes were upregulated by salt (200 mM) and ABA.

## 3. Discussion

The *Dof* family transcription factors, one of the most important family of transcriptional regulators in higher plants, are involved in many plant biological processes, such as plant growth, development, and response to abiotic stresses. *Dof* proteins are plant-specific transcription factors that play important roles in many physiological and biochemical processes [18]. The functions of *Dof* proteins have been previously studied in *Arabidopsis* [13], poplar [42], barley [43] and sorghum [44]. Nevertheless, the specific functions of most *Dof* genes in *C. songorica* are still unknown. In recent years, the complete *C. songorica* genome sequence was obtained by our research group [41], which provided a foundation to elucidate the *Dof* gene family structure and expression information in *C. songorica* under temperature, salt, and ABA treatment. In this study, we analyzed the gene structure, chromosomal location, conserved motifs, phylogenetic relationships, expression profile, and response to abiotic stress of *Dof* genes in *C. songorica*.

A comprehensive analysis in the genome of *C. songorica* was conducted and a multitude, a total of 50 *Dof* genes, were identified in the *C. songorica* genomic information, which is a much larger number than some species previous studied such as 27 in *B. distachyon* [14], 26 in moso bamboo [21], but much smaller than that in soybean [21], which contains 78 *Dof* gene members. The outcome suggested that the number of Dof TFs varies greatly among different species and the *CsDof* family genes may expand through different duplication events. Gene duplication is a mechanism for gene family expansion and new gene generation. In many plants, gene family expansion is primarily involved in segmental duplication, tandem duplication, and transposition events [45]. Syntentic analysis is usually used to identify the evolutionary relationship between genes. A previous study indicated that *C. songorica* experienced a whole-genome duplication event during biological evolution [21]. About 2/3 *CsDof* genes are syntentic, and most paralogous gene pairs existed on different chromosomes (Figure 2). The result is similar to gene families *CsNAC* and *bZIP*, and it has been reported that the expansion of *bZIP* and *CsNAC* gene families were produced by whole-genome duplication events [3,45]. These inferred that the expansion of the *C. songorica CsDof* gene family was also caused by whole-genome duplication events.

Previous studies have shown that *Dof* genes responded to cold, drought, ABA, and salt in different species, such as *Camellia sinensis* [46], *Phyllostachys edulis* [47], and potato [22]. For better understanding of how *CsDof* genes respond to abiotic stresses, high/low temperature, salt, and ABA treatments were used in this study. In bananas, 19 *MaDof* genes were not expressed under abiotic stress. In *C. songorica*, the *CsDof01*, *CsDof29*, *CsDof24*, and *CsDof48* did not show detectable expression (Table S5), which may indicate that these *CsDof* genes are pseudogenes or are expressed only under special conditions [18].

The diversity of gene structure is the basis for the evolution of multigene families [48]. The intron-exon divergence was closely related to the evolutionary relationship of plants [17]. The number of introns in *CsDof* genes ranged from 0 to 10, which was different from the results of previous studies. The number of introns in *Dof* genes ranged from 0 to 4 in banana [18], 0 to 2 in pepper [17], and 0 to 2 in sorghum [44]. These results showed that the *C. songorica Dof* gene family exhibited rich diversity. Moreover, the shared motifs were analyzed by the MEME search tool. In total, 20 motifs were identified in the 50 *Dof* proteins. Most of the *CsDof* proteins contained motif 1 or motif 3, which suggests that *CsDof* proteins have a highly conserved C2-C2 Zinc finger domain. Almost all *CsDof* proteins have motif 2. Motif 1, 3, and 2 together constituted conserved *Dof* domain (Figure 1, Figure 4 and Figure S1). In addition, combined with Figure S1, Figure 3 and Figure 4, we found most gene members in the same subfamily, which contain the similar motif compositions and shared similar exon/intron patterns in the light of lengths or intron numbers, such as *CsDof12* and *CsDof13*, *CsDof16* and *CsDof18*, *CsDof20* and *CsDof28*, *CsDof21* and *CsDof27*. This indicates that the *CsDof* genes have a conserved evolution and the proteins of the neighbouring subgroup may have similar functions. 

According to the amino acid sequences and phylogenetic relationships, the 50 *CsDof* genes were divided into four groups (Figure 4). Seventy-one percent of *CsDof* paralogous genes were in the same group, which verified the accuracy of phylogenetic tree construction (Figure 4; Table S2). For comparison with previous results, the *Arabidopsis* and *O. sativa Dof* genes were divided into four groups [13], and the sorghum *Dof* genes were divided into six groups [44]; the *Dof* genes in Chinese cabbage and soybean were also clustered into nine subgroups [21,22,31]. The differences might be because the *Dof* genes had different evolutionary models and characteristics in different species. Then phylogenetic tree was constructed based on the *Dof* genes of *C. songorica*, *A. thaliana*, *O. sativa*, and *B. distachyon*. As shown in Figure 5, several *C. songorica* and *A. thaliana*, *O. sativa* genes were clustered together with high bootstrap values, which implies that the *Dof* proteins of *C. songorica* and model plants have potential significant functional similarities. Interestingly, two groups (6 and 7) only included Gramineous (*C. songorica, O. sativa* and *B. distachyon*) members, and the same results are also found in wheat [49]. In addition, 77% of *C. songorica Dof* genes and their orthologous genes in *Arabidopsis* were in the same group, except for Gramineae unique groups (Figure 5; Tables S1 and S4), which verified the accuracy of the evolutionary tree constructed by four species.

The *Dof* family plays an important role in plant growth and development as well as in the response to abiotic stresses [31], as reported in previous studies [50,51]. In tomato, it has been demonstrated that the *SlCDF1-5*, which is the homologs of *Arabidopsis* CDFs, was induced in drought and salt stress response [30]. Most *StDof* genes were upregulated in drought, ABA, and high salinity in potato [22]. In *Triticum aestivum*, some *Dof* genes were reported to be regulated by salt and drought [20]. However, the response mechanisms to abiotic stresses of *CsDof* genes in *C. songorica* are still unknown. Therefore, researching the role of *Dofs* in *C. songorica* is necessary, especially in terms of drought, ABA, and high salinity. In addition, the gene expression profiles were studied according to RNA-seq data. As shown in Table S5 and Figure 6, some genes, which were divided into the same subgroup, showed similar expression profiles, such as *CsDof01* and *CsDof48*. Of course, there were also some genes in the same subgroup that had different expression profiles, such as *CsDof21* and *CsDof27*, *CsDof40*, and *CsDof22*. The result demonstrated that the *Dof* TFs is a representative of the plant-special transcription factor; similar conclusions were found in many reports—*Solanum melongena* L. [8], *Arabidopsis* [13], banana [18], and watermelon [52]. Here, six *CsDof* genes were selected for qPCR to detect expression profiles [31]. As shown in Figure 7, the expression profiles of these genes were consistent with the trend of transcriptome data (Figure S2). Furthermore, the expression of the selected genes was completely different for leaves and roots under same stress, such as *CsDof05* and *CsDof10* under ABA and low temperature. This result implied that the *CsDof* showed a specific and preferential expression in different tissues and stress (Figure 6 and Figure 7). Similar conclusions have been reported for the *StDof* genes of potato and *TaDof* genes of wheat [53,54]. The expression profiles of most *CsDof* genes implied that they can respond to abiotic stress. Some *CsDof* genes that were not expressed might have other functions and are only expressed in special tissues and conditions.

## 4. Materials and Methods

### 4.1. Plant Materials and Stress Treatments

Seeds of *C. songorica* were provided by Lanzhou University in China. After soaking the dried seeds in water for 24 h, the germinated seeds were grown under a light intensity of 200 μM mol photons m^−2^ s^−1^, a 16 h photoperiod, 30 °C day/28  °C night, and in monitored environments at 75–80% relative humidity. One-month old seedlings were treated with 40 °C (high temperature, HT), 4 °C (low temperature, LT), 50 mM (light salt stress, LSS), 100 mM NaCl (moderate salt stress, MSS), 200 mM NaCl (high salt stress, HSS), and ABA (100 uM) for 0 h (control, CK) and 24 h, and the leaves and root were harvested for RNA-seq [3,55]. For all treatments, plant materials from three biological replicates were harvested immediately, frozen in liquid nitrogen, and then stored at −80 °C until RNA isolation.

### 4.2. Identification and Structural Analysis of Dof Genes in C. songorica

First, the conserved *Dof* domain (PF02701) was downloaded from the Pfam database (Pfam 31.0, http://pfam.xfam.org accessed on 19 September 2019), according to its hidden Markov model (HMM). The *Dof* sequences of *Arabidopsis*, *O. sativa* and *B. distachyon* were downloaded from the database (https://phytozome.jgi.doe.gov/pz/portal.html accessed on 19 September 2019). The members of the *Dof* gene family were identified in the *C. songorica* genome sequence using BLAST 2.6.0 searches with the *Arabidopsis*, *O. sativa* and *B. distachyon Dof* sequences as a query (e-value cut-off >1 × 10^−5^) [41]. A conserved domain search of SMART (http://smart.embl-heidelberg.de/ accessed on 19 September 2019) was used to validate the identified *Dof* genes. The molecular weight (MW), theoretical isoelectric point (pI), and amino acid (aa) lengths of the *CsDof* proteins were calculated using ExPASy ProtParam online tools (http://web.expasy.org/protparam/ accessed on 19 September 2019) [56]. The orthologous genes of *C. songorica* in *Arabidopsis* were identified in an online website using the default setting (https://www.arabidopsis.org/Blast/index.jsp accessed on 19 September 2019). The subcellular localization of CsDof proteins were predicted by WoLF PSORT (https://wolfpsort.hgc.jp/ accessed on 19 September 2019).

### 4.3. Chromosomal Location and Gene Structure Analysis

The Tbtools v1.068 (https://github.com/CJ-Chen/TBtools accessed on 19 September 2019) software was used to locate each *CsDof* gene in the *C. songorica* chromosomes. The Gene Structure Display Server (GSDS) [57] (http://gsds.cbi.pku.edu.cn/ accessed on 19 September 2019) was used to create the *Dof* gene structures according to the primary sequence information obtained from the *C. songorica* genome database.

### 4.4. Conserved Motif and Phylogenetic Analysis

All *CsDof* protein-conserved motifs were analyzed using the MEME (Multiple Em for Motif Elicitation) (http://meme-suite.org/tools/meme accessed on 19 September 2019) v4.11.1 software online [58] according to the following parameters: select the site distribution: any number of repetitions; select the number of motifs: 20; the minimum and maximum sites of each motif were 5 and 100; and the minimum and maximum motif width were 6 and 100 [18]. Multiple sequence alignment of the *Dof* genes was performed using DNAMAN 7 software on the full protein sequences of *C. songorica*. The amino acid sequences of *C. songorica* and three other plants (*A. thaliana*, *O. sativa*, and *B. distachyon*) were aligned by Clustal X and the phylogenetic trees were constructed using the Neighbor-Joining (NJ) method of MEGA X, with the following parameters: poisson model, pairwise deletion, and 1000 bootstrap replications.

### 4.5. Gene Duplication and Syntenic Analysis

OrthoMCL soft V5 was used to identify the duplicated genes with default setting. The syntenic relationships of *CsDof* were illustrated with Tbtools v1.068. The Ka, Ks, and Ka/Ks values were calculated by the PAML yn00 NG model (http://abacus.gene.ucl.ac.uk/software/paml.html accessed on 19 September 2019). According to the λ value (6.5 × 10^−9^) of rice, the divergence time of *Dof* duplicated genes was calculated (T = Ks/2λ × 10^−6^ Mya) [59].

### 4.6. RNA-Seq and qPCR Analysis

The transcriptome data analysis process was the same as the previous research [3]. The heatmap of the *CsDof* genes expression profile was shown by the OmicShare online website (http://www.omicshare.com/tools accessed on 19 September 2019).

Total RNA was extracted from *C. songorica* after stress treatments for qPCR using RNAiso reagent (TaKaRa, Dalian, China). The extracted RNA was removed underlying genomic DNA and then reverse-transcribed into first-stand cDNA using the TaKaRa reagent Kit. qPCR was performed on an Applied Biosystems 7500 real-time PCR system (Applied Biosystems, Cheshire, UK) using a SYBR Green qPCR Kit (Sangon, Shanghai, China) according to the manufacturer’s protocol. qPCR was performed in a final volume of 20 μL containing 1 μL of cDNA, 10 μL of 2 × SG Fast qPCR Master Mix, 0.4 μL of forward primers, 0.4 μL of reverse primers (10 μM each), 2 μL of DNA Buffer, and 7.2 μL of double-distilled water. The qPCR amplification conditions were as follows: denaturation at 95 °C for 10 min, followed by 40 cycles of 95 °C for 15 s, and 60 °C for 1 min. As previously described, the expression level of each *CsDof* gene was calculated using the 2^−ΔΔCt^ method [60]. For the statistical analysis, SPSS 21.0 (Armonk, NY, USA) was used to detect the significant differences between the means (*p* < 0.05). The primers used for qPCR were designed using PerlPrimer v1.1.21 software with melting temperatures of 58–65 °C, lengths of 20–27 bp, and product lengths of 80–150 bp. The details were provided in Table S3. All primers were synthesized by Shanghai Sangon Biological Engineering Technology (Shanghai, China) [61].

## Data Availability

High temperature, low temperature, salt, and ABA treatment of *C. songorica* RNA-seq raw data were deposited under SRA accession numbers of SRP218434 and PRJNA634406. The *C. songorica* genome raw data and assembled genome sequences were deposited in NCBI (PRJNA634005) and National Genomics Data Center (PRJCA002752), respectively.

## Supplementary Materials

The following are available online at https://www.mdpi.com/article/10.3390/plants10050850/s1, Figure S1: Sequence logos of *Dof* domains in *C. songorica* obtained using the MEME program. The total height of the stack represents the level of sequence conservation. The height of the residue in the stack represents the relative frequency of each residue in that position. Figure S2: The correlation of real-time PCR analysis and RNA-Seq (FPKM). Table S1: *Dof* genes identified and characterized in *C. songorica*. Table S2: Inference of duplication time of *Dof* paralogous pairs in *C. songorica.* Table S3: Sequences of primers. Table S4: List of *Dof* genes of *A. thaliana, O. sativa, B. distachyon.* Table S5: The expression amount of *CsDof* genes in *C. songorica.*

## References

[1] Qu L.-J., Zhu Y.-X. (2006). Transcription factor families in Arabidopsis: Major progress and outstanding issues for future research-Commentary. Curr. Opin. Plant Biol..

[2] Yu Q., Li C., Zhang J., Tian Y., Wang H., Zhang Y., Zhang Z., Xiang Q., Han X., Zhang L. (2020). Genome-wide identification and expression analysis of the Dof gene family under drought stress in tea (*Camellia sinensis*). PeerJ.

[3] Yan Q., Wu F., Ma T., Zong X., Ma Q., Li J., Zhao Y., Wang Y., Zhang J. (2019). Comprehensive analysis of bZIP transcription factors uncovers their roles during dimorphic floret differentiation and stress response in *Cleistogenes songorica*. BMC Genom..

[4] Wasserman W.W., Sandelin A. (2004). Applied bioinformatics for the identification of regulatory elements. Nat. Rev. Genet..

[5] Yanagisawa S. (1997). Dof DNA-binding domains of plant transcription factors contribute to multiple protein-protein interactions. Eur. J. Biochem..

[6] Nakano T., Suzuki K., Ohtsuki N., Tsujimoto Y., Fujimura T., Shinshi H. (2006). Identification of genes of the plant-specific transcription-factor families cooperatively regulated by ethylene and jasmonate in *Arabidopsis thaliana*. J. Plant Res..

[7] Yanagisawa S. (2002). The Dof family of plant transcription factors. Trends Plant Sci..

[8] Wei Q., Wang W., Hu T., Hu H., Bao C. (2018). Genome-wide identification and characterization of Dof transcription factors in eggplant (*Solanum melongena* L.). PeerJ.

[9] Wen C.L., Cheng Q., Zhao L., Mao A., Yang J., Yu S., Weng Y., Xu Y. (2016). Identification and characterisation of Dof transcription factors in the cucumber genome. Sci. Rep..

[10] Chen M., Liu X., Huan L., Sun M., Liu L., Chen X., Gao D., Li L. (2017). Genome-wide analysis of Dof family genes and their expression during bud dormancy in peach (*Prunus persica*). Sci. Hortic..

[11] Zou Z., Zhang X. (2019). Genome-wide identification and comparative evolutionary analysis of the Dof transcription factor family in physic nut and castor bean. PeerJ.

[12] Yanagisawa S. (2004). Dof Domain Proteins: Plant-specific transcription factors associated with diverse phenomena unique to plants. Plant Cell Physiol..

[13] Lijavetzky D., Carbonero P., Vicente-Carbajosa J. (2003). Genome-wide comparative phylogenetic analysis of the rice and Arabidopsis Dof gene families. BMC Evol. Biol..

[14] Sara H.A., Virginia G.C., Pilar C., Cristina B.S. (2012). The family of DOF transcription factors in *Brachypodium distachyon*: Phylogenetic comparison with rice and barley DOFs and expression profiling. BMC Plant Biol..

[15] Min G.E., Yuan-Da L., Tan L.I., Zhang T.F., Zhang X.L., Zhao H. (2014). Genome-wide identification and analysis of Dof transcription factor family in maize. Sci. Agric. Sin..

[16] Zou Z., Yang J., Zhang X. (2019). Insights into genes encoding respiratory burst oxidase homologs (RBOHs) in rubber tree (*Hevea brasiliensis Muell*. Arg.). Ind. Crops Prod..

[17] Wu Z., Cheng J., Cui J., Xu X., Liang G., Luo X., Chen X., Tang X., Hu K., Cheng Q. (2016). Genome-wide identification and expression profile of Dof transcription factor gene family in pepper (*Capsicum annuum*. L.). Front. Plant Sci..

[18] Dong C., Hu H., Xie J. (2016). Genome-wide analysis of the DNA-binding with one zinc finger (Dof) transcription factor family in bananas. Genome.

[19] Shu Y.J., Song L.L., Zhang J., Liu Y., Guo C.H. (2015). Genome-wide identification and characterization of the Dof gene family in *Medicago truncatula*. Genet. Mol. Res..

[20] Shaw L., Mcintyre C., Gresshoff P., Xue G. (2009). Members of the Dof transcription factor family in *Triticum aestivum* are associated with light-mediated gene regulation. Funct. Integr. Genom..

[21] Guo Y., Qiu L.J. (2013). Genome-wide analysis of the Dof transcription factor gene family reveals soybean-specific duplicable and functional characteristics. PLoS ONE.

[22] Venkatesh J., Park S.W. (2015). Genome-wide analysis and expression profiling of DNA-binding with one zinc finger (Dof) transcription factor family in potato. Plant Physiol. Biochem..

[23] Wang T., Yue J.J., Wang X.J., Xu L., Li L.B., Gu X.P. (2016). Genome-wide identification and characterization of the Dof gene family in moso bamboo (*Phyllostachys heterocycla* var. *pubescens*). Genes Genom..

[24] Yanagisawa S. (2000). Dof1 and Dof2 transcription factors are associated with expression of multiple genes involved in carbon metabolism in maize. Plant J..

[25] Fornara F., Panigrahi K.C., Gissot L., Sauerbrunn N., Rühl M., Jarillo J.A., Coupland G. (2009). Arabidopsis DOF transcription factors act redundantly to reduce CONSTANS expression and are essential for a photoperiodic flowering response. Dev. Cell.

[26] Kumar R., Taware R., Gaur V.S., Guru S., Kumar A. (2009). Influence of nitrogen on the expression of TaDof1 transcription factor in wheat and its relationship with photo synthetic and ammonium assimilating efficiency. Mol. Biol. Rep..

[27] Le Hir R., Bellini C. (2013). The plant-specific Dof transcription factors family: New players involved in vascular system development and functioning in Arabidopsis. Front. Plant Sci..

[28] Kumar A., Kanwal P., Gupta A.K., Singh B., Gaur V.S. (2014). A full-length Dof1 transcription factor of finger millet and its response to a circadian cycle. Plant Mol. Biol. Report..

[29] Zou H.-F., Zhang Y.-Q., Wei W., Chen H.-W., Song Q.-X., Liu Y.-F., Zhao M.-Y., Wang F., Zhang B.-C., Lin Q. (2013). The transcription factor AtDOF4. 2 regulates shoot branching and seed coat formation in Arabidopsis. Biochem. J..

[30] Corrales A.-R., Nebauer S.G., Carrillo L., Fernández-Nohales P., Marqués J., Renau-Morata B., Granell A., Pollmann S., Vicente-Carbajosa J., Molina R.-V. (2014). Characterization of tomato Cycling Dof Factors reveals conserved and new functions in the control of flowering time and abiotic stress responses. J. Exp. Bot..

[31] Ma J., Li M.-Y., Wang F., Tang J., Xiong A.-S. (2015). Genome-wide analysis of Dof family transcription factors and their responses to abiotic stresses in Chinese cabbage. BMC Genom..

[32] Imaizumi T., Schultz T.F., Harmon F.G., Ho L.A., Kay S.A. (2005). FKF1 F-box protein mediates cyclic degradation of a repressor of CONSTANS in Arabidopsis. Science.

[33] Skirycz A., Radziejwoski A., Busch W., Hannah M.A., Czeszejko J., Kwaśniewski M., Zanor M., Lohmann J.U., De Veylder L., Witt I. (2010). The DOF transcription factor OBP1 is involved in cell cycle regulation in *Arabidopsis thaliana*. Plant J..

[34] Ward J.M., Cufr C.A., Denzel M.A., Neff M.M. (2005). The Dof transcription factor OBP3 modulates phytochrome and cryptochrome signaling in Arabidopsis. Plant Cell.

[35] Yang Q., Chen Q., Zhu Y., Li T. (2017). Identification of *MdDof* genes in apple and analysis of their response to biotic or abiotic stress. Funct. Plant Biol..

[36] Yan Q., Wu F., Yan Z., Li J., Ma T., Zhang Y., Zhao Y., Wang Y., Zhang J. (2019). Differential co-expression networks of long non-coding RNAs and mRNAs in *Cleistogenes songorica* under water stress and during recovery. BMC Plant Biol..

[37] Yu X., Wang Y., Zeng Y., Su D. (2004). Effects of temperature and osmotic potential on seed germination of *Cleistogenes songorica* and *Plantago lessingii*. Acta Ecol. Sin..

[38] Kong L., Zhang J., Liu Z., Wang Y. (2013). Cloning of a S-adenosyl methionine synthetase gene from *Cleistogenes songorica* and its expression under drought stress. Acta Prataculturae Sin..

[39] Zhang J., Kong L., Liu Z., Jahufer Z., Duan Z., Huo Y., Di H., Wang Y. (2015). Stress-induced expression in Arabidopsis with a Dehydrin LEA protein from *Cleistogenes songorica*, a xerophytic desert grass. Plant Omics.

[40] Zhang J., Duan Z., Jahufer Z., An S., Wang Y. (2014). Stress-inducible expression of a Cleistogenes songorica ALDH gene enhanced drought tolerance in transgenic. Plant Omics.

[41] Zhang J., Wu F., Yan Q., John U., Cao M., Xu P., Zhang Z., Ma T., Zong X., Li J. (2021). The genome of *Cleistogenes songorica* provides a blueprint for functional dissection of dimorphic flower differentiation and drought adaptability. Plant Biotechnol. J..

[42] Yang X., Tuskan G.A., Cheng M.Z. (2006). Divergence of the Dof gene families in poplar, Arabidopsis, and rice suggests Multiple modes of gene evolution after duplication. Plant Physiol..

[43] Morenorisueno M.Á., Martínez M., Vicentecarbajosa J., Carbonero P. (2007). The family of DOF transcription factors: From green unicellular algae to vascular plants. Mol. Genet. Genom..

[44] Kushwaha H., Gupta S., Singh V.K., Rastogi S., Yadav D. (2011). Genome wide identification of Dof transcription factor gene family in sorghum and its comparative phylogenetic analysis with rice and Arabidopsis. Mol. Biol. Rep..

[45] Zong X., Yan Q., Wu F., Ma Q., Zhang J. (2020). Genome-wide analysis of the role of NAC family in flower development and abiotic stress responses in *Cleistogenes songorica*. Genes.

[46] Li H., Huang W., Liu Z.W., Wang Y.X., Zhuang J. (2016). Transcriptome-based analysis of Dof family transcription factors and their responses to abiotic stress in tea plant (*Camellia sinensis*). Int. J. Genom..

[47] Liu J., Cheng Z., Xie L., Li X., Gao J. (2019). Multifaceted role of PheDof12-1 in the regulation of flowering time and abiotic stress responses in moso bamboo (*Phyllostachys edulis*). Int. J. Mol. Sci..

[48] Cao J., Shi F., Liu X., Huang G., Zhou M. (2010). Phylogenetic analysis and evolution of aromatic amino acid hydroxylase. FEBS Lett..

[49] Fang Z., Jiang W., He Y., Ma D., Liu Y., Wang S., Zhang Y., Yin J. (2020). Genome-wide identification, structure characterization, and expression profiling of Dof transcription factor gene family in wheat (*Triticum aestivum* L.). Agronomy.

[50] Noguero M., Atif R.M., Ochatt S., Thompson R.D. (2013). The role of the DNA-binding One Zinc Finger (DOF) transcription factor family in plants. Plant Sci..

[51] Gupta S., Malviya N., Kushwaha H., Nasim J., Bisht N.C., Singh V., Yadav D. (2015). Insights into structural and functional diversity of Dof (DNA binding with one finger) transcription factor. Planta.

[52] Zhou Y., Cheng Y., Wan C., Li J., Yang Y., Chen J. (2020). Genome-wide characterization and expression analysis of the Dof gene family related to abiotic stress in watermelon. PeerJ.

[53] Liu Y., Liu N., Deng X., Liu D., Li M., Cui D., Hu Y., Yan Y. (2020). Genome-wide analysis of wheat DNA-binding with one finger (Dof) transcription factor genes: Evolutionary characteristics and diverse abiotic stress responses. BMC Genom..

[54] Cai X., Zhang Y., Zhang C., Zhang T., Hu T., Ye J., Zhang J., Wang T., Li H., Ye Z. (2013). Genome-wide analysis of plant-specific Dof transcription factor family in Tomato. J. Integr. Plant Biol..

[55] Yan Q., Zong X., Wu F., Li J., Ma T., Zhao Y., Ma Q., Wang P., Wang Y., Zhang J. (2020). Integrated analysis of co-expression, conserved genes and gene families reveal core regulatory network of heat stress response in *Cleistogenes songorica*, a xerophyte perennial desert plant. BMC Genom..

[56] Gasteiger E., Hoogland C., Gattiker A., Duvaud S.E., Wilkins M.R., Appel R.D., Bairoch A. (2005). Protein identification and analysis tools on the ExPASy server. The Proteomics Protocols Handbook.

[57] Guo A.Y., Zhu Q.H., Chen X., Luo J.C. (2007). GSDS: A gene structure display server. Hereditas.

[58] Bailey T.L., Boden M., Buske F.A., Frith M., Grant C.E., Clementi L., Ren J., Li W.W., Noble W.S. (2009). MEME SUITE: Tools for motif discovery and searching. Nucleic Acids Res..

[59] Han F., Zhu B. (2011). Evolutionary analysis of three gibberellin oxidase genesin rice, Arabidopsis, and soybean. Gene.

[60] Zhang J.Y., Wang Y.R., Nan Z.B. (2009). Relative and absolute quantification expression analysis of *CsSAMDC* gene as a case. China Biotechnol..

[61] Zhu W., Lee Y.S. (2004). Dexel-based force-torque rendering and volume updating for 5-DOF haptic product prototyping and virtual sculpting. Comput. Ind..

