# Supplementary material for: Genome-Wide Analysis and Expression Profiles of the Dof Family in Cleistogenes songorica under Temperature, Salt and ABA Treatment
Source: Plants (Basel). 2021 Apr 23;10(5):850. doi: 10.3390/plants10050850 (PMC8146245; doi:10.3390/plants10050850)
Supplement: Supplementary file 1 [file plants-10-00850-s001.zip › supplementary information/Table S1.docx]

**Table S1 *Dof* genes identified and characterized in *Cleistogenes songorica***

| Gene name | CCG_id | Location | CDS length (bp) | Protein length (aa) | Number of amino acids | Molecular weight | Theoretical pI | At ortholog locus | Dof Domain | Putative localization |
| --- | --- | --- | --- | --- | --- | --- | --- | --- | --- | --- |
| *CsDof01* | CCG038870.1 | Chr5: 42198318 : 42199214 | 596 | 197 | 193 | 20762.63 | 9.21 | AT4G24060.1 | 1-36 | chloroplast |
| *CsDof02* | CCG011842.1 | Chr5: 49547996 : 49548817 | 842 | 279 | 273 | 27840.51 | 8.84 | AT3G61850.4 | 76-133 | nucleus |
| *CsDof03* | CCG006814.1 | Chr7: 3028167 : 3029237 | 1097 | 364 | 356 | 37263.73 | 11.06 | AT5G65590.1 | 61-119 | chloroplast |
| *CsDof04* | CCG003010.1 | Chr7: 9615639 : 9617089 | 1026 | 341 | 333 | 35423 | 10.08 | AT1G28310.2 | 24-62 | chloroplast |
| *CsDof05* | CCG040769.1 | Chr5: 56000959 : 56002053 | 1121 | 372 | 364 | 37373.27 | 9.19 | AT5G60850.1 | 74-132 | nucleus |
| *CsDof06* | CCG012627.1 | Chr5: 58487728 : 58489185 | 1248 | 415 | 405 | 41017.35 | 9.08 | AT5G02460.1 | 112-170 | nucleus |
| *CsDof07* | CCG017664.2 | Chr5: 37331839 : 37332930 | 1118 | 371 | 363 | 36575.75 | 8.64 | AT5G60200.1 | 48-106 | mitochondria |
| *CsDof08* | CCG013893.1 | Chr5: 32002836 : 32004951 | 1328 | 441 | 431 | 45710.99 | 7.83 | AT5G39660.2 | 99-157 | nucleus |
| *CsDof09* | CCG009370.1 | Chr7: 21233130 : 21234479 | 1156 | 383 | 375 | 38746.09 | 9.43 | AT5G02460.1 | 84-142 | nucleus |
| *CsDof10* | CCG026591.1 | Chr13: 41247856 : 41255456 | 2289 | 762 | 744 | 78418.04 | 7.05 | AT2G25430.1 | 83-141 | nucleus |
| *CsDof11* | CCG045958.1 | Chr12: 15325341 : 15326328 | 881 | 292 | 286 | 29758.26 | 5.41 | AT3G52440.1 | 8-49 | chloroplast |
| *CsDof12* | CCG034995.1 | Chr13: 24289057 : 24289608 | 564 | 187 | 183 | 19303.62 | 10.41 | AT2G34140.1 | 63-121 | nucleus |
| *CsDof13* | CCG021870.1 | Chr12: 17932229 : 17932858 | 644 | 213 | 209 | 22444.08 | 9.53 | AT3G47500.1 | 68-126 | chloroplast |
| *CsDof14* | CCG016384.1 | Chr3: 24535111 : 24535788 | 694 | 229 | 225 | 23180.57 | 9.57 | AT1G07640.2 | 36-94 | chloroplast |
| *CsDof15* | CCG009876.1 | Chr3: 11351677 : 11352915 | 1269 | 422 | 412 | 44245.24 | 5.24 | AT3G52440.1 | 119-177 | chloroplast |
| *CsDof16* | CCG019196.1 | Chr3: 1377671 : 1378126 | 466 | 153 | 151 | 15650.62 | 10.06 | AT3G47500.1 | 66-125 | mitochondria |
| *CsDof17* | CCG015561.1 | Chr8: 47636019 : 47636732 | 730 | 241 | 237 | 25737.31 | 9.67 | AT2G34140.1 | 97-155 | chloroplast，mitochondria |
| *CsDof18* | CCG032447.1 | Chr8: 44285932 : 44286396 | 475 | 156 | 154 | 15901.93 | 10.28 | AT3G47500.1 | 70-128 | nucleus，mitochondria |
| *CsDof19* | CCG009494.1 | Chr3: 18259047 : 18261902 | 1583 | 526 | 514 | 56073.4 | 5.22 | AT5G39660.2 | 161-218 | nucleus |
| *CsDof20* | CCG037995.1 | Chr18: 15592543 : 15593316 | 792 | 263 | 257 | 26230.92 | 9.58 | AT2G28810.2 | 49-107 | nucleus |
| *CsDof21* | CCG029300.1 | Chr18: 14929849 : 14931108 | 999 | 332 | 324 | 33603.67 | 9.03 | AT5G02460.1 | 99-156 | nucleus |
| *CsDof22* | CCG020969.1 | Chr6: 4890907 : 4893509 | 1213 | 402 | 394 | 39931.02 | 9.25 | AT4G24060.1 | 91-149 | nucleus |
| *CsDof23* | CCG001904.1 | Chr6: 28093954 : 28098922 | 1847 | 614 | 600 | 64718.65 | 6.26 | AT5G39660.2 | 222-280 | nucleus |
| *CsDof24* | CCG045370.1 | Chr6: 23386519 : 23391334 | 421 | 138 | 136 | 14628.54 | 6.07 | AT1G07640.2 | 31-85 | mitochondria |
| *CsDof25* | CCG045273.1 | Chr10: 666336 : 667061 | 744 | 247 | 241 | 25850.12 | 8.47 | AT5G66940.1 | 88-146 | nucleus |
| *CsDof26* | CCG035618.1 | Chr0: 5421688 : 5424294 | 907 | 300 | 294 | 30747.91 | 8.32 | AT5G62940.1 | 58-116 | nucleus |
| *CsDof27* | CCG038129.1 | Chr19: 378741 : 380036 | 1022 | 339 | 331 | 33995.94 | 8.89 | AT3G55370.3 | 100-157 | nucleus |
| *CsDof28* | CCG038208.1 | Chr19: 1064851 : 1065642 | 810 | 269 | 263 | 26702.39 | 9.74 | AT2G28810.2 | 57-114 | nucleus |
| *CsDof29* | CCG025844.1 | Chr5: 27128340 : 27129257 | 940 | 311 | 305 | 31808.79 | 9.05 | AT5G65590.1 | 34-92 | nucleus |
| *CsDof30* | CCG011214.1 | Chr16: 15654668 : 15662860 | 2238 | 745 | 727 | 77967.83 | 8.62 | AT1G69010.1 | 531-589 | nucleus |
| *CsDof31* | CCG042927.1 | Chr11: 22197197 : 22197964 | 786 | 261 | 255 | 26378.39 | 8.43 | AT3G50410.1 | 38-96 | nucleus |
| *CsDof32* | CCG022106.1 | Chr2: 6347127 : 6351670 | 1293 | 430 | 420 | 46306.49 | 9 | AT4G24060.1 | 199-257 | nucleus |
| *CsDof33* | CCG003764.1 | Chr4: 59657007 : 59658675 | 1343 | 446 | 436 | 45616.98 | 9.96 | AT1G51700.1 | 248-306 | chloroplast |
| *CsDof34* | CCG021988.1 | Chr1: 25510313 : 25511359 | 1073 | 356 | 348 | 34453.32 | 8.15 | AT5G60200.1 | 39-97 | nucleus |
| *CsDof35* | CCG008302.1 | Chr1: 5404532 : 5406989 | 715 | 236 | 232 | 25953.92 | 10.81 | AT5G62940.1 | 72-130 | nucleus,cytoplasm |
| *CsDof36* | CCG046388.1 | Chr0: 4593150 : 4594013 | 884 | 293 | 287 | 29834.16 | 9.22 | AT5G60850.1 | 47-105 | chloroplast,nucleus |
| *CsDof37* | CCG007324.1 | Chr1: 6654954 : 6656012 | 1085 | 360 | 352 | 35664.6 | 8.96 | AT4G24060.1 | 61-119 | nucleus |
| *CsDof38* | CCG051733.1 | Chr1: 32763983 : 32767040 | 1361 | 452 | 442 | 46720.5 | 6.59 | AT5G39660.2 | 125-183 | chloroplast |
| *CsDof39* | CCG048161.2 | Chr9: 18272724 : 18273598 | 768 | 255 | 249 | 25345.39 | 9.5 | AT2G46590.2 | 88-145 | chloroplast |
| *CsDof40* | CCG012234.1 | Chr10: 20098062 : 20100496 | 872 | 289 | 283 | 29912.82 | 10.58 | AT4G24060.1 | 124-182 | nucleus |
| *CsDof41* | CCG005273.1 | Chr10: 24614020 : 24614706 | 617 | 204 | 200 | 21474.99 | 8.23 | AT5G66940.1 | 48-106 | chloroplast，nucleus |
| *CsDof42* | CCG024650.1 | Chr15: 12664909 : 12665952 | 1070 | 355 | 347 | 34254.14 | 8.79 | AT1G28310.2 | 35-93 | nucleus |
| *CsDof43* | CCG047096.1 | Chr17: 35561225 : 35562824 | 813 | 270 | 264 | 28147.86 | 9.45 | AT4G24060.1 | 47-105 | nucleus |
| *CsDof44* | CCG029025.1 | Chr14: 1941892 : 1942590 | 715 | 236 | 232 | 24573.45 | 9.28 | AT4G24060.1 | 33-91 | nucleus |
| *CsDof45* | CCG029651.1 | Scaffold_36: 47063 : 47959 | 830 | 275 | 269 | 27122.19 | 9.34 | AT2G46590.1 | 100-157 | chloroplast |
| *CsDof46* | CCG050595.1 | Scaffold_827: 104492 : 105418 | 979 | 314 | 308 | 32337.07 | 5.79 | AT3G52440.1 | 33-91 | nucleus |
| *CsDof47* | CCG024152.1 | Scaffold_28919: 7922 : 10120 | 1453 | 482 | 472 | 50470.57 | 8.15 | AT5G39660.2 | 146-204 | nucleus |
| *CsDof48* | CCG047655.1 | Chr19: 67945 : 68842 | 807 | 268 | 262 | 28751.76 | 10.1 | AT4G24060.1 | 40-83 | nucleus |
| *CsDof49* | CCG012384.1 | Chr4: 83067 : 89083 | 1370 | 455 | 445 | 48537.29 | 9.31 | AT4G24060.1 | 230-288 | plas |
| *CsDof50* | CCG051458.1 | Scaffold_865: 12059 : 12988 | 952 | 315 | 309 | 31292.02 | 8.17 | AT5G65590.1 | 36-92 | nucleus |
