# Supplementary material for: Genome-Wide Analysis and Expression Profiles of the Dof Family in Cleistogenes songorica under Temperature, Salt and ABA Treatment
Source: Plants (Basel). 2021 Apr 23;10(5):850. doi: 10.3390/plants10050850 (PMC8146245; doi:10.3390/plants10050850)
Supplement: Supplementary file 1 [file plants-10-00850-s001.zip › supplementary information/Table S2.docx]

**Table S2 Inference of duplication time of Dof paralogous pairs in *Cleistogenes songorica***

| Paralogous pairs | *ka* | *ks* | *ka/ks* | Data (million years ago) |
| --- | --- | --- | --- | --- |
| *CsDof02/CsDof20* | 0.3817 | 1.4406 | 0.265 | 110.82 |
| *CsDof02/CsDof04* | 0.0718 | 0.3628 | 0.198 | 27.91 |
| *CsDof05/CsDof03* | 0.1175 | 0.3866 | 0.3039 | 29.74 |
| *CsDof05/CsDof13* | 0.5314 | 2.9431 | 0.1806 | 226.39 |
| *CsDof05/CsDof21* | 0.6868 | 3.8188 | 0.1798 | 293.75 |
| *CsDof05/CsDof20* | 0.6234 | 2.3735 | 0.2627 | 182.58 |
| *CsDof06/CsDof09* | 0.4795 | 1.543 | 0.3108 | 118.69 |
| *CsDof10/CsDof09* | 0.5619 | 2.3929 | 0.2348 | 184.07 |
| *CsDof10/CsDof06* | 0.5124 | 2.3655 | 0.2166 | 181.96 |
| *CsDof12/CsDof13* | 0.0973 | 0.3053 | 0.3187 | 23.48 |
| *CsDof12/CsDof32* | 0.6051 | 3.5666 | 0.1697 | 274.35 |
| *CsDof12/CsDof17* | 0.4503 | 3.4252 | 0.1315 | 263.48 |
| *CsDof12/CsDof16* | 0.3831 | 2.1313 | 0.1797 | 163.95 |
| *CsDof13/CsDof17* | 0.5158 | 1.6081 | 0.3208 | 123.70 |
| *CsDof14/CsDof05* | 0.5565 | 2.2343 | 0.2491 | 171.87 |
| *CsDof14/CsDof24* | 0.3652 | 1.9168 | 0.1905 | 147.45 |
| *CsDof16/CsDof13* | 0.3587 | 1.6326 | 0.2197 | 125.58 |
| *CsDof16/CsDof17* | 0.4339 | 1.3385 | 0.3242 | 102.96 |
| *CsDof16/CsDof18* | 0.0703 | 0.3001 | 0.2343 | 23.08 |
| *CsDof17/CsDof18* | 0.4421 | 1.0896 | 0.4057 | 83.82 |
| *CsDof19/CsDof23* | 0.0298 | 0.1172 | 0.2544 | 9.02 |
| *CsDof20/CsDof04* | 0.4268 | 2.351 | 0.1815 | 180.85 |
| *CsDof20/CsDof03* | 0.6613 | 2.2961 | 0.288 | 176.62 |
| *CsDof25/CsDof08* | 0.6387 | 3.6764 | 0.1737 | 282.80 |
| *CsDof25/CsDof32* | 0.5898 | 2.3277 | 0.2534 | 179.05 |
| *CsDof25/CsDof43* | 0.6259 | 3.509 | 0.1784 | 269.92 |
| *CsDof26/CsDof35* | 0.25 | 0.6868 | 0.364 | 52.83 |
| *CsDof27/CsDof21* | 0.0144 | 0.3534 | 0.0408 | 27.18 |
| *CsDof28/CsDof02* | 0.378 | 1.3067 | 0.2893 | 100.52 |
| *CsDof28/CsDof03* | 0.7634 | 1.996 | 0.3825 | 153.54 |
| *CsDof28/CsDof04* | 0.4848 | 2.1489 | 0.2256 | 165.30 |
| *CsDof28/CsDof05* | 0.6851 | 2.0102 | 0.3408 | 154.63 |
| *CsDof28/CsDof20* | 0.0723 | 0.3762 | 0.1921 | 28.94 |
| *CsDof31/CsDof30* | 0.0433 | 0.3463 | 0.1249 | 26.64 |
| *CsDof31/CsDof33* | 0.262 | 2.2952 | 0.1141 | 176.55 |
| *CsDof33/CsDof30* | 0.5975 | 2.4505 | 0.2438 | 188.50 |
| *CsDof36/CsDof30* | 0.4671 | 3.5331 | 0.1322 | 271.78 |
| *CsDof36/CsDof31* | 0.4734 | 2.4864 | 0.1904 | 191.26 |
| *CsDof36/CsDof33* | 0.5184 | 3.6039 | 0.1438 | 277.22 |
| *CsDof40/CsDof37* | 0.2538 | 1.353 | 0.1876 | 104.08 |
| *CsDof44/CsDof43* | 0.1007 | 0.2875 | 0.3504 | 22.12 |
