# Supplementary material for: Genome-Wide Analysis and Expression Profiles of the Dof Family in Cleistogenes songorica under Temperature, Salt and ABA Treatment
Source: Plants (Basel). 2021 Apr 23;10(5):850. doi: 10.3390/plants10050850 (PMC8146245; doi:10.3390/plants10050850)
Supplement: Supplementary file 1 [file plants-10-00850-s001.zip › supplementary information/TableS3.docx]

**Table S3 The sequences of primers**

| gene name | Forward sequence(F) | Reverse sequence(R) |
| --- | --- | --- |
| *CsDof05* | CGTCAGATCCCTGCTACAC | GATGACGGTGGGAGCTTTA |
| *CsDof10* | GAATGCCAACAAAGAGGGAAGC | GTTCATCCAGGTCTGTGGCA |
| *CsDof23* | GGATGAGCATGAGAAAGGAGTC | TCTTCTGCATTGTTCATCTGCT |
| *CsDof25* | CAGCTGCTAGATTCCTCTTCTG | GTCAGTGGGCATGACGATAG |
| *CsDof34* | ACGACCGCCTACGAGAT | CTCCTGCATCCACGGTTT |
| *CsDof37* | GAGCACAACAAAGATCAAGGAG | CACCATTGCTACTGCCATTC |
