# Supplementary material for: Genome-Wide Analysis and Expression Profiles of the Dof Family in Cleistogenes songorica under Temperature, Salt and ABA Treatment
Source: Plants (Basel). 2021 Apr 23;10(5):850. doi: 10.3390/plants10050850 (PMC8146245; doi:10.3390/plants10050850)
Supplement: Supplementary file 1 [file plants-10-00850-s001.zip › supplementary information/TableS4.docx]

**Table S4 List of Dof genes of *A. thaliana*, *O. sativa*, *B. distachyon***

|  | | | | | |
| --- | --- | --- | --- | --- | --- |
| gene name | locus | gene name | locus | gene name | locus |
| *AtDof1* | AT1G07640 | *OsDof1* | Os07g48570 | *BdDof1* | Bradi1g03710 |
| *AtDof2* | AT1G21340 | *OsDof2* | Os01g55340 | *BdDof2* | Bradi5g18640 |
| *AtDof3* | AT1G26790 | *OsDof3* | Os01g15900 | *BdDof3* | Bradi3g38980 |
| *AtDof4* | AT1G28310 | *OsDof4* | Os01g17000 | *BdDof4* | Bradi2g10640 |
| *AtDof5* | AT1G29160 | *OsDof5* | Os03g16850 | *BdDof5* | Bradi2g37130 |
| *AtDof6* | AT1G47655 | *OsDof6* | Os03g07360 | *BdDof6* | Bradi1g26570 |
| *AtDof7* | AT1G51700 | *OsDof7* | Os10g26620 | *BdDof7* | Bradi1g14570 |
| *AtDof8* | AT1G64620 | *OsDof8* | Os05g36900 | *BdDof8* | Bradi2g49440 |
| *AtDof9* | AT1G69570 | *OsDof9* | Os01g64590 | *BdDof9* | Bradi2g55980 |
| *AtDof10* | AT2G28510 | *OsDof10* | Os07g32510 | *BdDof10* | Bradi2g24040 |
| *AtDof11* | AT2G28810 | *OsDof11* | Os02g47810 | *BdDof11* | Bradi1g73710 |
| *AtDof12* | AT2G34140 | *OsDof12* | Os02g49440 | *BdDof12* | Bradi3g52880 |
| *AtDof13* | AT2G37590 | *OsDof13* | Os03g38870 | *BdDof13* | Bradi3g29940 |
| *AtDof14* | AT2G46590 | *OsDof14* | Os06g17410 | *BdDof14* | Bradi2g62380 |
| *AtDof15* | AT3G21270 | *OsDof15* | Os01g09720 | *BdDof15* | Bradi1g07600 |
| *AtDof16* | AT3G45610 | *OsDof16* | Os04g47990 | *BdDof16* | Bradi2g09720 |
| *AtDof17* | AT3G47500 | *OsDof17* | Os02g45200 | *BdDof17* | Bradi2g50370 |
| *AtDof18* | AT3G50410 | *OsDof18* | Os08g38220 | *BdDof18* | Bradi4g04260 |
| *AtDof19* | AT3G52440 | *OsDof19* | Os03g42200 | *BdDof19* | Bradi2g19930 |
| *AtDof20* | AT3G55370 | *OsDof20* | Os01g48290 | *BdDof20* | Bradi3g25670 |
| *AtDof21* | AT3G61850 | *OsDof21* | Os12g38200 | *BdDof21* | Bradi1g15420 |
| *AtDof22* | AT4G00940 | *OsDof22* | Os07g13260 | *BdDof22* | Bradi1g17410 |
| *AtDof23* | AT4G21030 | *OsDof23* | Os02g15350 | *BdDof23* | Bradi3g51510 |
| *AtDof24* | AT4G21040 | *OsDof24* | Os05g02150 | *BdDof24* | Bradi5g26240 |
| *AtDof25* | AT4G21050 | *OsDof25* | Os04g58190 | *BdDof25* | Bradi1g66600 |
| *AtDof26* | AT4G21080 | *OsDof26* | Os10g35300 | *BdDof26* | Bradi4g33000 |
| *AtDof27* | AT4G24060 | *OsDof27* | Os12g39990 | *BdDof27* | Bradi2g46300 |
| *AtDof28* | AT4G38000 | *OsDof28* | Os03g55610 |  |  |
| *AtDof29* | AT5G02460 | *OsDof29* | Os03g60630 |  |  |
| *AtDof30* | AT5G39660 | *OsDof30* | Os09g29960 |  |  |
| *AtDof31* | AT5G60200 |  |  |  |  |
| *AtDof32* | AT5G60850 |  |  |  |  |
| *AtDof33* | AT5G62430 |  |  |  |  |
| *AtDof34* | AT5G62940 |  |  |  |  |
| *AtDof35* | AT5G65590 |  |  |  |  |
| *AtDof36* | AT5G66940 |  |  |  |  |
