# Supplementary material for: Genome-Wide Analysis and Expression Profiles of the Dof Family in Cleistogenes songorica under Temperature, Salt and ABA Treatment
Source: Plants (Basel). 2021 Apr 23;10(5):850. doi: 10.3390/plants10050850 (PMC8146245; doi:10.3390/plants10050850)
Supplement: Supplementary file 1 [file plants-10-00850-s001.zip › supplementary information/TableS5.docx]

**Table S5 The expression amount of *CsDof* genes in *C. songorica***

| gene name | CK_L | HT_L | LT_L | ABA_L | LSS_L | MSS_L | HSS_L | CK_R | HT_R | LT_R | ABA_R | LSS_R | MSS_R | HSS_R |
| --- | --- | --- | --- | --- | --- | --- | --- | --- | --- | --- | --- | --- | --- | --- |
| *CsDof01* | 0 | 0 | 0 | 0 | 0 | 0 | 0 | 0 | 0 | 0 | 0 | 0 | 0 | 0 |
| *CsDof02* | 0 | 0.020007 | 0.156898 | 0 | 0 | 0.902819 | 0.070248 | 0.071787 | 0 | 0.047367 | 0.044842 | 0 | 0 | 0 |
| *CsDof03* | 0.174938 | 0.013419 | 0.219045 | 0 | 0 | 1.132799 | 0.334283 | 0.923313 | 0.384192 | 1.445782 | 1.588245 | 1.834879 | 2.51035 | 1.16064 |
| *CsDof04* | 0.028759 | 0 | 0.307615 | 0 | 0 | 0.793934 | 0.142316 | 0 | 0 | 0 | 0 | 0.013994 | 0 | 0.046959 |
| *CsDof05* | 0.052106 | 0.099178 | 0.441256 | 0 | 0 | 0.519136 | 0.081509 | 0.845114 | 0.363651 | 0.492366 | 1.499055 | 0.551801 | 1.35948 | 0.317889 |
| *CsDof06* | 2.274593 | 0.785606 | 2.017569 | 3.946292 | 3.794988 | 5.6554 | 3.267807 | 3.146195 | 2.849941 | 3.799991 | 3.269909 | 2.937081 | 4.727663 | 1.751439 |
| *CsDof07* | 0.693378 | 0.173647 | 0.267566 | 0.951969 | 1.241655 | 1.48282 | 1.155925 | 0 | 0 | 0.055129 | 0 | 0 | 0.016737 | 0.189967 |
| *CsDof08* | 34.02531 | 90.89892 | 114.5761 | 53.9648 | 77.04631 | 16.08622 | 24.72041 | 15.38928 | 30.12183 | 177.6107 | 9.862605 | 12.69854 | 9.471519 | 8.494135 |
| *CsDof09* | 1.651056 | 2.212743 | 2.524424 | 1.624283 | 1.774198 | 2.567118 | 1.535395 | 10.87212 | 12.92322 | 21.04702 | 5.135826 | 21.32339 | 9.810428 | 8.14361 |
| *CsDof10* | 6.446389 | 2.101139 | 10.72133 | 4.108596 | 4.498624 | 13.84751 | 8.269679 | 6.117834 | 2.315677 | 6.972064 | 6.240387 | 6.273845 | 6.429531 | 3.328591 |
| *CsDof11* | 7.187468 | 0 | 5.313438 | 0.363821 | 3.731043 | 12.63455 | 10.72402 | 0.051435 | 0.125292 | 0.140706 | 0.252135 | 0 | 0.170385 | 0.019201 |
| *CsDof12* | 1.176703 | 0.800432 | 0.103578 | 0.602265 | 1.156523 | 2.5902 | 1.789415 | 4.00681 | 1.071361 | 2.736385 | 12.747 | 1.171707 | 3.148605 | 0.524788 |
| *CsDof13* | 6.225973 | 0.098141 | 8.517175 | 0.409613 | 5.734129 | 6.919416 | 5.555177 | 3.97665 | 0.308624 | 1.81699 | 0.818441 | 0.414874 | 0.444654 | 1.590574 |
| *CsDof14* | 1.412567 | 0.894163 | 3.19762 | 3.13009 | 3.77033 | 3.312785 | 4.444765 | 4.315405 | 6.086487 | 1.27182 | 4.03791 | 34.97037 | 13.92069 | 43.7912 |
| *CsDof15* | 1.692887 | 0.03169 | 1.154791 | 0.031785 | 0.460892 | 1.893675 | 2.01174 | 0.34338 | 0.021578 | 0.244792 | 1.718615 | 0.101403 | 0.711813 | 0.15549 |
| *CsDof16* | 7.03076 | 75.08387 | 9.601716 | 4.406397 | 1.855227 | 13.57006 | 8.26423 | 3.886752 | 38.86221 | 10.67319 | 5.288338 | 4.674761 | 5.348806 | 9.479265 |
| *CsDof17* | 7.871227 | 9.957557 | 7.85248 | 0.694385 | 1.967844 | 21.60775 | 7.02402 | 7.682225 | 94.99983 | 9.73925 | 8.207305 | 5.415548 | 5.915795 | 7.901832 |
| *CsDof18* | 3.435387 | 71.4092 | 1.762199 | 1.80105 | 1.197621 | 4.660295 | 3.25783 | 4.733525 | 24.46663 | 4.252545 | 4.932065 | 4.927123 | 3.973715 | 7.54148 |
| *CsDof19* | 1.10238 | 6.354013 | 13.54019 | 5.401745 | 3.36546 | 0.974437 | 1.672656 | 2.292205 | 1.481437 | 15.9589 | 1.524225 | 10.60777 | 3.50365 | 7.60768 |
| *CsDof20* | 1.369726 | 0.021132 | 4.459671 | 0.457255 | 0.185893 | 15.04456 | 2.323357 | 0.293948 | 0 | 0.763095 | 0 | 0 | 0 | 0 |
| *CsDof21* | 3.468773 | 0.520548 | 7.3969 | 1.555125 | 2.72525 | 14.36739 | 5.79928 | 1.41764 | 0.562192 | 3.218535 | 2.794205 | 1.707437 | 2.407715 | 1.28014 |
| *CsDof22* | 23.22582 | 2.666314 | 9.71308 | 25.99452 | 31.97657 | 38.1453 | 15.13922 | 69.40595 | 20.111 | 41.22107 | 63.45375 | 110.0329 | 68.82641 | 94.07679 |
| *CsDof23* | 7.292237 | 4.943225 | 53.28417 | 19.29869 | 12.03269 | 3.565219 | 6.688451 | 2.753436 | 2.285212 | 44.81948 | 2.818765 | 5.541095 | 2.570244 | 4.134317 |
| *CsDof24* | 0 | 0 | 0 | 0 | 0 | 0 | 0 | 0 | 0 | 0 | 0 | 0.156214 | 0 | 0.2929 |
| *CsDof25* | 4.026383 | 0.701332 | 5.354525 | 2.223525 | 1.552617 | 3.866295 | 0.733732 | 11.09795 | 9.312687 | 19.93655 | 5.935065 | 7.473023 | 8.87497 | 2.323855 |
| *CsDof26* | 5.29086 | 0.939555 | 8.68396 | 3.239747 | 2.973799 | 10.77825 | 9.038677 | 2.354417 | 1.247308 | 3.71704 | 4.333787 | 2.345819 | 4.445661 | 2.753518 |
| *CsDof27* | 39.78087 | 174.5787 | 61.0276 | 45.67425 | 66.16855 | 55.7198 | 69.37205 | 169.755 | 196.532 | 92.0656 | 85.0391 | 110.1046 | 104.9458 | 84.34445 |
| *CsDof28* | 0.427349 | 4.052456 | 1.487102 | 0 | 0 | 1.545175 | 0.626156 | 0 | 0.029225 | 0.111505 | 0 | 0 | 0 | 0.043094 |
| *CsDof29* | 0 | 0 | 0 | 0 | 0 | 0 | 0 | 0 | 0 | 0 | 0 | 0 | 0 | 0 |
| *CsDof30* | 14.72036 | 8.482456 | 40.74026 | 19.14214 | 16.21725 | 14.40413 | 14.41816 | 9.238552 | 5.929922 | 73.26424 | 17.0328 | 14.83588 | 11.60379 | 20.78327 |
| *CsDof31* | 134.9032 | 77.37559 | 92.18991 | 162.363 | 127.7051 | 140.6015 | 192.701 | 50.48819 | 25.67204 | 106.4094 | 75.83647 | 159.9211 | 103.4457 | 141.7538 |
| *CsDof32* | 0 | 0.03801 | 0 | 0 | 0 | 0 | 0 | 0.013575 | 0 | 0 | 0 | 0.032267 | 0.013795 | 0 |
| *CsDof33* | 8.205297 | 0.459553 | 3.554731 | 15.76582 | 15.9021 | 10.78539 | 10.74065 | 12.85964 | 2.691632 | 4.868089 | 12.41696 | 12.80867 | 10.92973 | 15.23985 |
| *CsDof34* | 40.53896 | 8.236933 | 31.21621 | 40.14605 | 35.1115 | 44.6057 | 28.03292 | 53.3051 | 21.25929 | 130.4508 | 38.0853 | 70.66017 | 74.6312 | 42.4639 |
| *CsDof35* | 2.882959 | 1.239061 | 4.888118 | 1.490803 | 0.76533 | 9.028738 | 4.666196 | 1.125714 | 2.210985 | 2.870132 | 3.024847 | 1.001354 | 4.657939 | 1.582284 |
| *CsDof36* | 13.58535 | 0.555016 | 8.093335 | 46.3486 | 19.73705 | 6.666285 | 15.15781 | 0.407458 | 0.527421 | 0.125365 | 0.826797 | 0.970677 | 0.283863 | 0.951619 |
| *CsDof37* | 8.60655 | 2.021567 | 12.86249 | 5.9448 | 7.77283 | 22.24155 | 6.546555 | 56.6882 | 6.909867 | 64.72055 | 33.44965 | 75.273 | 67.4529 | 88.2476 |
| *CsDof38* | 21.64684 | 3.623008 | 13.27222 | 59.05003 | 53.62146 | 8.056976 | 14.12829 | 9.259924 | 7.628684 | 6.041075 | 6.803816 | 23.78573 | 15.15776 | 9.28414 |
| *CsDof39* | 5.371368 | 2.280707 | 3.317934 | 3.782959 | 5.084902 | 13.00911 | 7.311628 | 34.4759 | 9.030117 | 18.79009 | 21.6425 | 55.45801 | 47.64495 | 61.25581 |
| *CsDof40* | 0.362204 | 0.307713 | 0.882335 | 0.303326 | 0.641212 | 0.409753 | 0.577591 | 2.306664 | 1.144752 | 1.369126 | 1.199308 | 1.576173 | 1.032002 | 1.37603 |
| *CsDof41* | 1.461153 | 0.278227 | 3.36294 | 1.748405 | 1.58261 | 4.390865 | 3.107095 | 5.18662 | 6.780063 | 3.251255 | 12.74694 | 15.35317 | 25.26645 | 8.890085 |
| *CsDof42* | 54.16322 | 11.22293 | 22.01436 | 56.88569 | 56.79027 | 58.04331 | 37.38987 | 57.39665 | 47.17226 | 75.8441 | 46.86275 | 83.0315 | 84.4722 | 59.5143 |
| *CsDof43* | 0.093867 | 0 | 0 | 0 | 0.042391 | 0.297559 | 0 | 0.260908 | 0.028873 | 0.045041 | 0.04866 | 0.152791 | 0.160679 | 0.270269 |
| *CsDof44* | 0 | 0 | 0 | 0 | 0 | 0.150585 | 0.179422 | 0.103597 | 0.036019 | 0 | 0.085997 | 0.144401 | 0.119231 | 0.071736 |
| *CsDof45* | 8.88855 | 9.533971 | 20.83542 | 5.79357 | 8.074317 | 17.05181 | 13.95739 | 35.4649 | 10.5875 | 132.4817 | 19.71675 | 48.37107 | 37.61601 | 43.57225 |
| *CsDof46* | 18.00643 | 0.13352 | 30.99751 | 0.217029 | 8.34976 | 28.11925 | 22.4504 | 1.378944 | 0.050513 | 1.27692 | 0.468455 | 0.055752 | 0.046763 | 1.997375 |
| *CsDof47* | 15.717 | 9.10501 | 35.49735 | 49.97895 | 29.73905 | 11.83115 | 16.50782 | 10.21801 | 18.5828 | 77.75165 | 15.05325 | 38.55967 | 23.52225 | 43.46635 |
| *CsDof48* | 0 | 0 | 0 | 0 | 0 | 0 | 0 | 0 | 0 | 0 | 0 | 0 | 0 | 0 |
| *CsDof49* | 1.219255 | 110.894 | 0.90587 | 0.2402 | 0.381737 | 0.255657 | 0.263992 | 1.461074 | 2.767645 | 4.122985 | 0.578091 | 0.543443 | 2.306692 | 1.032846 |
| *CsDof50* | 0.674812 | 0 | 0.056781 | 0.061889 | 0.080331 | 0.208382 | 0.108165 | 0 | 0 | 0 | 0 | 0 | 0 | 0 |
