# Supplementary figures and images for: Genome-Wide Analysis and Expression Profiles of the Dof Family in Cleistogenes songorica under Temperature, Salt and ABA Treatment
Source: Plants (Basel). 2021 Apr 23;10(5):850. doi: 10.3390/plants10050850 (PMC8146245; doi:10.3390/plants10050850)

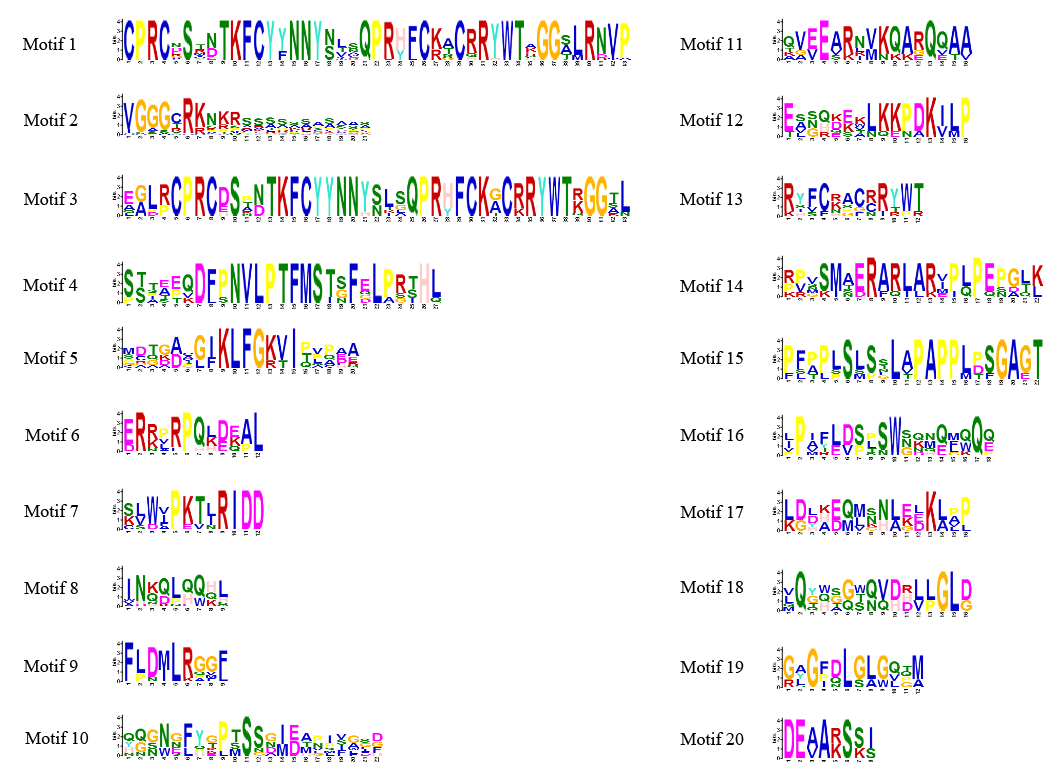

Supplement: Supplementary file 1 [file plants-10-00850-s001.zip › supplementary information/Fig. S1.tif]

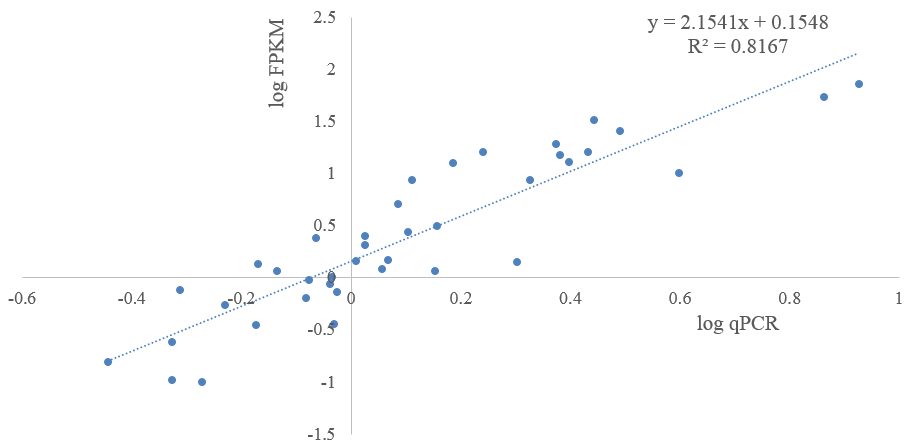

Supplement: Supplementary file 1 [file plants-10-00850-s001.zip › supplementary information/Fig. S2.tiff]
